# US smokers' reactions to a brief trial of oral nicotine products

**DOI:** 10.1186/1477-7517-8-1

**Published:** 2011-01-10

**Authors:** Richard J O'Connor, Kaila J Norton, Maansi Bansal-Travers, Martin C Mahoney, K Michael Cummings, Ron Borland

**Affiliations:** 1 Department of Health Behavior, Roswell Park Cancer Institute, Buffalo, NY, USA; 2 Cancer Council Victoria, Melbourne, Australia

## Abstract

**Background:**

It has been suggested that cigarette smokers will switch to alternative oral nicotine delivery products to reduce their health risks if informed of the relative risk difference. However, it is important to assess how smokers are likely to use cigarette alternatives before making predictions about their potential to promote individual or population harm reduction.

**Objectives:**

This study examines smokers' interest in using a smokeless tobacco or a nicotine replacement product as a substitute for their cigarettes.

**Methods:**

The study included 67 adult cigarette smokers, not currently interested in quitting, who were given an opportunity to sample four alternative oral nicotine products: 1) Camel Snus, 2) Marlboro Snus, 3) Stonewall dissolvable tobacco tablets, and 4) Commit nicotine lozenges. At visit 1, subjects were presented information about the relative benefits/risks of oral nicotine delivery compared to cigarettes. At visit 2, subjects were given a supply of each of the four products to sample at home for a week. At visit 3, subjects received a one-week supply of their preferred product to see if using such products reduced or eliminated cigarette use.

**Results:**

After multiple product sampling, participants preferred the Commit lozenges over the three smokeless tobacco products (p = 0.011). Following the one week single-product trial experience, GEE models controlling for gender, age, level of education, baseline cigarettes use, and alternative product chosen, indicated a significant decline in cigarettes smoked per day across one week of single-product sampling (p < 0.01, from 11.8 to 8.7 cigarettes per day), but no change in alternative product use (approximately 4.5 units per day). Biomarkers of exposure showed no change in cotinine, but a 19% reduction in exhaled CO (p < 0.001).

**Conclusions:**

Findings from this study show that smokers, who are currently unwilling to make a quit attempt, may be willing to use alternative products in the short term as a temporary substitute for smoking. However, this use is more likely to be for partial substitution (i.e. they will continue to smoke, albeit at a lower rate) rather than complete substitution. Of the various substitutes offered, smokers were more willing to use a nicotine replacement product over a tobacco-based product.

## Background

While the harms of tobacco smoking have been well documented for decades, more than 20% of US adults continue to smoke[1]. This seeming lack of progress has led to interest in harm reduction as a complementary tobacco control strategy, particularly with products that offer reduced toxicity to individual users[2]. Two products that have received substantial discussion as potential harm reduction options are smokeless tobacco (particularly low-toxin forms such as Swedish snus) and nicotine replacement therapy (medicinal nicotine), which is currently approved only for limited duration use in smoking cessation[3-7]. However, the promotion of reduced harm products, especially smokeless tobacco, remains controversial. Commonly expressed concerns include a lack of reliable data on health risk reduction (as opposed to exposure), impacts on smoking behavior (e.g., dual use and continued nicotine dependence), and the potential attractiveness of products to former and non-users of tobacco, especially youth[2,8-11]. Further complicating matters is the fact that since 2005, major cigarette manufacturers have, either through partnership or acquisition, moved into the smokeless tobacco business. This has allowed tobacco manufacturers to introduce smokers to new smokeless tobacco products for use in situations where smoking is restricted[12-14].

Consumer perceptions and responses are key components to understanding the adoption and maintenance of new and modified tobacco products[15]. The Family Smoking Prevention and Tobacco Control Act of 2009 (FSPTCA) empowers the FDA to regulate tobacco products by considering consumer perceptions in decisions about regulations "appropriate for the protection of public health"[16]. So, knowing what users believe about products, and their reactions to those products, are recognized to be important to understanding how smokeless tobacco and medicinal nicotine are likely to be used and thus their potential as substitutes for cigarettes. However, independent research has been limited in this area. Timberlake [17] found only 13% of California smokers were receptive to using smokeless tobacco instead of smoking. Studies outside North America have found that 34% of New Zealand smokers and 48% of Australian smokers were interested in trying smokeless tobacco[18,19]. Interest in switching from cigarettes to an alternative form of nicotine delivery may depend on smokers' preexisting beliefs about alternative products. Data consistently show that consumers incorrectly believe nicotine causes cancer,[20-22] and that smokeless tobacco products are as, if not more, dangerous than cigarettes[23-26]. So, the information presented about products may be an important factor in influencing interest. Shiffman and colleagues [27] presented smokers with descriptions of both medicinal nicotine and tobacco-based products positioned as smoking substitutes, and found that smokers generally preferred the medicinal nicotine products to the tobacco-based products. On the other hand, Heavner and colleagues[28] surveyed smokers in Edmonton, Canada, and reported that 75% were willing to try a hypothetical product carrying 99% less risk than smoking. However, neither of these studies involved direct experience.

Recently, Carpenter and Gray [29] reported that, compared to continued smoking, use of Ariva or Stonewall compressed tobacco lozenges reduced cigarette consumption and increased intentions to quit. A series of studies by Schneider and colleagues [30-32] examined preferences among different nicotine replacement products, concluding that individuals have varied reactions to different nicotine delivery modes, and sampling of treatments can identify key reactions that predict later quitting success. Caldwell and colleagues [33] found that, among heavy smokers given two-weeks experience, snus or Zonnic oral nicotine sachet were preferred over nicotine gum. Cobb and colleagues [34] using a series of laboratory sessions with smokers, showed that non-combustible products (Ariva, Camel Snus, Marlboro Snus, Commit) delivered less nicotine than smokers usual brand of cigarettes and failed to suppress tobacco abstinence symptoms as effectively as cigarettes. Overall, the literature suggests that it is important to try to assess smokers' reactions to proposed cigarette alternatives before making predictions about their potential to promote harm reduction relative to continued smoking.

The present study was designed to examine smokers' interest in using a smokeless tobacco (SL) or nicotine replacement product (NR) as a substitute for their cigarettes. Specifically, we set out to address three questions: 1) Among various options, which alternative source of oral nicotine delivery do smokers prefer? 2) When given an opportunity to use their preferred product, how would they use it (i.e., complete or partial substitution)? 3) Would brief *ad libitum *use of the oral nicotine substitute alter exposure to cigarettes as assessed by CPD, carbon monoxide and nicotine levels?

## Methods

### Study Participants

Recruitment occurred via community flyers and advertisements in local newspapers, which sought smokers interested in trying alternative tobacco and nicotine products. Participants were eligible if they smoked ten or more cigarettes per day for at least one year, were not currently using any other nicotine or tobacco product, were able to read and write in English, had no medical contraindications (e.g., heart disease) for nicotine replacement use, had not made a quit attempt in the previous 30 days, and were not planning to quit in the next 30 days. Sixty-seven participants met eligibility criteria, of whom 44 completed the entire study. Demographics for those who did or did not complete the study are show in Table 1. Overall, only prior use of NRT significantly differentiated the completers from those lost to follow-up.

**Table 1:** Demographic characteristics of study participants (N = 67).

Variable	Levels	Lost to follow-up	Completed Study	p-value*
		(n = 23)	(n = 44)	
Gender	Female	52.247.8	52.3	0.994
	Male		47.7	

Highest Level of Education	Less than HS	39.1	20.5	0.053
	HS Graduate	34.8	22.7	
	More than HS	26.1	56.8	

Age	< 40	30.4	18.2	0.379
	41 - 54	60.9	63.6	
	55 +	8.7	18.2	

Race	White	56.5	67.4	0.183
	Black	43.5	25.6	
	Other	0	7	

Intention to Quit in the next year	No	8.7	15.9	0.411
	Yes	91.3	84.1	

How addicted do you consider yourself?	Very	78.3	72.7	0.274
	Somewhat	17.4	27.3	
	Not at all	4.3	0	

Prior use of NR	Yes	26.1	52.3	0.04
	No	73.9	47.7	

Prior use of ST	Yes	8.7	11.488.6	0.735
	No	91.3		

Cigarettes per Day	Mean	21.4	22.2	0.519
	(SE)	-3.7	-1.9	

Minutes to First Cigarette after Waking	Median	5	5	0.662
	(IQR)	(2-15)	(5-15)	

* categorical variables compared by chi-square; continuous variables compared by Wilcoxon test, p-value < 0.05

HS = High school

### Study design

**Figure 1:**
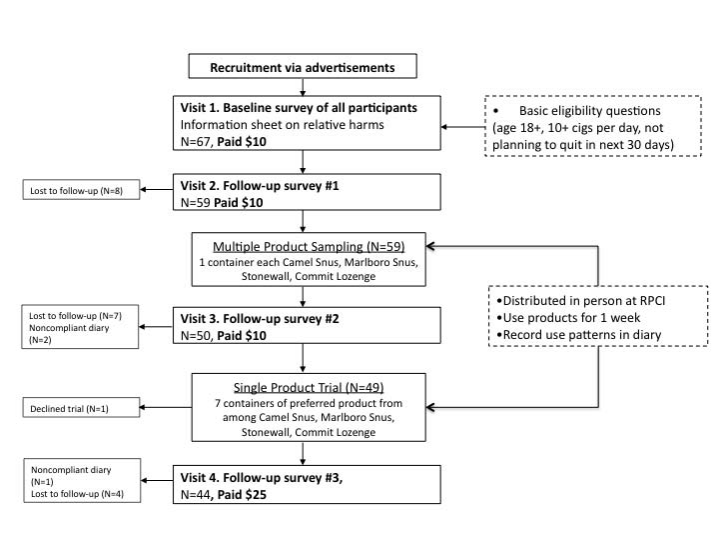
**Flowchart detailing study involvement of participants**.

Participants visited the laboratory for four separate sessions (each one week apart) between June and December 2008, as part of a broader study of the effects of information on knowledge of tobacco and nicotine harms (Borland et al., in preparation). Figure 1 outlines the study course for participants. Sessions 1 and 2 presented information about the relative risks of smokeless tobacco and nicotine replacement products compared to cigarettes to provide a health-based rationale for considering these products as alternatives. The findings related to knowledge will not be presented here. At the end of Session 2, after completing a questionnaire, participants were offered the opportunity to sample four different SL and NR products (Multiple-Product Sampling). Participants were given one package of each of four SL/NR products (detailed below), with instructions to use each product at least once and then to use as much of these products as they wished over the following week. Baseline carbon monoxide (CO) and saliva samples (e.g, salivary cotinine) were obtained at this time. One week later, participants returned for Session 3, completed a questionnaire about their experiences with the four trial products and were given the opportunity to select their preferred SL/NR product out of the four trial products and use it for one more week (Single-Product Trial). Participants were provided with this additional week-long supply of their chosen product at no cost and asked to record their usage patterns in a daily diary. They were asked to bring all of their tins and any unused portions to their next lab visit to confirm self-reported usage. One week later, participants completed Session 4, consisting of a follow-up questionnaire and collection of CO and saliva samples. Participants received $10 per visit for completion of the first 3 visits and $25 for completion of the fourth visit for a maximum possible compensation of $55. The study protocol was approved by the Roswell Park Cancer Institute Institutional Review Board, and all participants provided documented informed consent prior to participation.

### Products

All test products were purchased on the open market in 2008 and previous studies have examined characteristics of these or similar products[35-37]. At the end of Session 2, participants were provided with samples of three smokeless tobacco products [one container each of Camel Snus, 20 pouches; Marlboro Snus, 12 pouches; and Stonewall Hard Snuff, 20 tablets] and one package of oral nicotine replacement product (Commit^® ^Lozenge, 24 4 mg lozenges). The smokeless products contained varying amounts of free nicotine - from 0.7 mg/g (Marlboro Snus Mint) to 1.5 mg/g (Stonewall) to 6.4 mg/g (Camel Snus Frost)[36,37]. For standardization purposes, all products were offered only in their 'mint' versions, so that the availability of specific flavors would not drive selections. At the end of Session 3, participants selected one of these four products to use for an additional week and received 7 containers/packages of that product.

### Measures

Questionnaire items focused on the specific SL/NR products offered and their experiences with the products, including which product overall they liked most/least, willingness to use the products instead of smoking under a range of scenarios, and interest in actually continuing to use such products as a partial or complete replacement for cigarettes. Participants were also asked a series of questions pertaining to their willingness to pay for the smokeless and nicotine replacement products. Daily diaries were used to track numbers of cigarettes and substitute products used each day. At baseline participants self-reported their usual number of cigarettes per day and time to first cigarette after waking, which were recoded into the Heavy Smoking Index (HSI) [38]. Exhaled CO was tested using a Micro 4 Smokerlyzer (Bedfont, UK) using standard protocols. Cotinine in saliva was tested using the EIA method by an outside laboratory (Salimetrics LLC, University Park, PA).

### Data analysis

All analyses were performed using SPSS 16.0 (Chicago, IL). Descriptive statistics and frequencies were used to initially characterize the data. Kendall's tau-b was used to examine concordance between product rankings. Change in responses over time was examined using paired t-tests. Cotinine values were transformed using the natural logarithm to normalize the distribution prior to analysis. Generalized estimating equations (GEE) with log link and first-order autoregressive working correlation matrix were applied to examine daily patterns of product use; a normal distribution best fit models of cigarette use, while a gamma distribution best fit models of alternative product use. Statistical significance was accepted at a p-value of <0.05.

## Results

### Multiple-product sampling

Of the initial 67 participants, 59 remained in the study to sample products. However, seven were lost to follow-up and two were excluded because they did not use the provided products. Table 2 presents information on use of each product and opinions related to each product as assessed at Session 3. Participants did not use a large amount (between 10% and 20%) of each product supplied, and they appear to have distributed their product usage approximately equally across all products. However, their post-sampling choices were non-random, with Commit lozenge the most well-liked and Stonewall the least liked products (Kendall's tau-b = -0.314, p = 0.011). Further, participants believed Camel Snus to contain the most nicotine, while Commit was considered to have the least. No relationship was observed between perceived nicotine content and positive product rating (Kendall's tau-b = -0.119, p = 0.338) or negative product rating (Kendall's tau-b = 0.024, p = 0.832). There were no significant associations between the preferred product and participant gender, age, intention to quit smoking, HSI score, or ever use of ST or NR products.

**Table 2:** Alternative product preferences (N = 50).

	Camel Snus	Marlboro Snus	Stonewall	Commit 4 mg	
Proportion of supplied units used (Median, IQR)	10	16.7	10	12.5	χ^2^(3) = 589.31
	(5-25)	(8-58)	(5-28)	(4-33)	p = 0.003 ^a^

Proportion of all products used (Median, IQR)	20	23.4	22.2	25	χ^2^(3) = 142.2
	(9-25)	(9-35)	(8-33)	(15-50)	p = 0.524 ^a^

					χ^2^(3) = 005.31
Product Liked Most (%)	12.5	31.2	12.5	43.8	p = 0.004 ^b^

					χ^2^(3) = 333.8
Product Liked Least (%)	27.1	10.4	39.6	22.9	p = 0.040 ^b^

					χ^2^(3) = 519.8
Product believed to contain most nicotine (%)	42.6	21.3	23.4	12.8	p = 0.030 ^b^

					χ^2^(3) = 944.31
Product selected for one-week trial (%)^c^	14.3	28.6	12.2	44.9	p = 0.004 ^b^

IQR = interquartile range

a. Friedman test; b. One-sample chi-square test

c. One participant declined to use any product for the one-week trial.

#### Patterns of use

**Figure 2:**
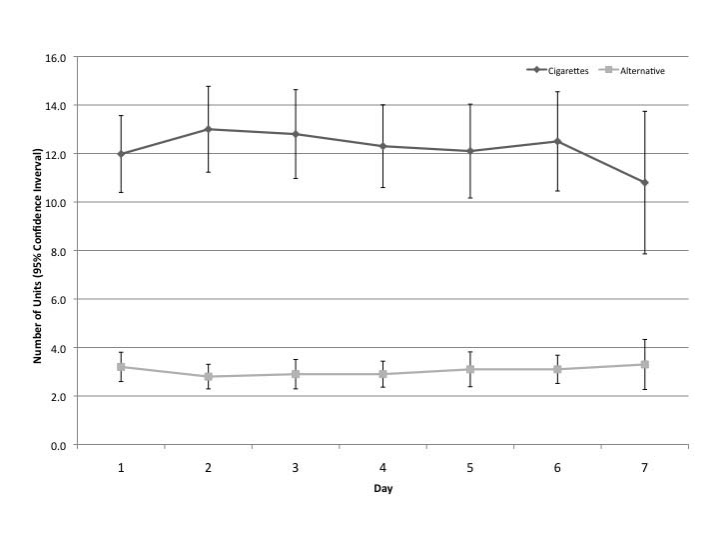
**Alternative product versus cigarette patterns of use during multiple-product sampling (N = 50) phase**.

As illustrated in Figure 2, over the course of the seven-day sampling phase, both cigarette use (12.0 cigarettes on day 1 to 10.8 cigarettes on day 7; p = 0.509) and alternative product use (3.2 units on day 1 to 3.3 units on day 7; p = 0.512) were fairly consistent. This was confirmed in GEE models controlling for gender, age (categorized as <40, 41-54, ≥55 years), level of education, Heavy Smoking Index score, and alternative product most preferred, where we observed no significant effect of day for cigarettes and alternative products (see Table 3). HSI score was positively associated with cigarette use over the sampling week, but not with alternative product use.

**Table 3:** Parameter Estimates from GEE modeling of cigarette and alternative product use during multiple product sampling and single-product trial phases.

	Cigarettes				Alternative Products			
	**B**	**SE**	**Wald χ^2 ^(df = 1)**	**p**	**B**	**SE**	**Wald χ^2 ^(df = 1)**	**p**

**MULTIPLE PRODUCT SAMPLING PHASE**								

Intercept	1.966	0.354	30.911	<.001	0.682	0.486	1.969	0.161

Day	-0.012	0.019	0.436	0.509	-0.015	0.022	0.43	0.512

Female (ref = Male)	0.046	0.104	0.193	0.66	-0.029	0.185	0.024	0.877

Less than HS (ref = More than HS)	0.044	0.117	0.143	0.705	**-0.454**	**0.177**	**6.611**	**0.01**

HS graduate (ref = More than HS)	-0.08	0.149	0.287	0.592	-0.204	0.188	1.173	0.279

40 and under (ref = 55 and older)	-0.186	0.186	1.003	0.317	0.036	0.193	0.034	0.854

41-54 (ref = 55 and older)	-0.169	0.14	1.457	0.227	0.139	0.203	0.468	0.494

Camel Snus (ref = Stonewall)	-0.135	0.187	0.524	0.469	**0.489**	**0.228**	**4.608**	**0.032**

Marlboro Snus (ref = Stonewall)	-0.154	0.145	1.131	0.288	0.418	0.234	3.19	0.074

Commit (ref = Stonewall)	-0.274	0.158	3.006	0.083	**0.515**	**0.216**	**5.685**	**0.017**

HSI	**0.211**	**0.049**	**18.591**	**<.001**	0.022	0.071	0.092	0.762

**SINGLE PRODUCT TRIAL PHASE**								
Intercept	1.762	0.471	13.987	<.001	0.414	0.526	0.62	0.431

Day	-0.039	**0.014**	**8.186**	**0.004**	-0.006	0.023	0.08	0.777

Female (ref = Male)	-0.128	0.145	0.787	0.375	-0.084	0.177	0.227	0.634

Less than HS (ref = More than HS)	**0.344**	**0.139**	**6.307**	**0.012**	**-0.568**	**0.181**	**9.855**	**0.002**

HS graduate (ref = More than HS)	-0.071	0.131	0.295	0.587	-0.026	0.206	0.016	0.898

40 and under (ref = 55 and older)	-0.443	**0.192**	**5.31**	**0.021**	-0.283	0.266	1.134	0.287

41-54 (ref = 55 and older)	-0.264	0.154	3.299	0.069	0.044	0.216	0.041	0.839

Camel Snus (ref = Stonewall)	0.16	0.203	0.617	0.432	0.091	0.283	0.102	0.749

Marlboro Snus (ref = Stonewall)	-0.18	0.171	1.109	0.292	0.358	0.312	1.312	0.252

Commit (ref = Stonewall)	0.007	0.19	0.001	0.97	0.49	0.263	3.467	0.063

HSI	0.245	**0.061**	**16.386**	**<.001**	**0.234**	**0.081**	**8.265**	**0.004**

**NOTE**: HSI = Heavy Smoking Index

Parameter estimates in bold indicate statistically significant effects.

### Single-product trial

Of the 50 participants who sampled multiple products, 49 selected a preferred product to use for one additional week, while one declined to use any products. Of these, five did not complete the final visit. As expected, choice of product following the one week trial period closely mirrored reports of which product participants liked most after multiple-product sampling (Kendall's tau-b = 0.907, p < 0.001), with 14% choosing Camel Snus, 29% choosing Marlboro Snus, 12% choosing Stonewall, and 45% choosing Commit Lozenge. While no overall significant effects by product were observed, Table 4 suggests participants who preferred Camel Snus anticipated using it *in addition *to cigarettes, moreso than those preferring other alternative products. However, they differed significantly in the median amount they were willing to pay for a container/package of that product, from a low of $2 for Stonewall to $5 for Commit Lozenge, with substantial inter-individual variability. 80% of participants reported that they were very or somewhat likely to purchase their preferred product in the next year.

**Table 4:** Participant likelihood to use, purchase, & pay for alternative products.(N = 44)

	Preferred product (Overall)	Camel Snus	Marlb Snus	Stone-wall	Commit 4 mg	Test/p-value*
Likely to use *instead of *cigarettes (%)						

Very	20.5	14.3	15.4	0	31.6	χ^2^(6) = 96.4

Somewhat	45.5	42.9	46.2	40	47.4	p = 0.584

Not at all	34.1	42.9	38.5	60	21.1	

Likely to use *in addition to *cigarettes (%)						

Very	47.7	71.4	23.1	40	57.9	χ^2^(6) = 52.6

Somewhat	38.6	28.6	53.8	40	31.6	p = 0.396

Not at all	13.6	0	23.1	20	10.5	

Likely to purchase in next year (%)						

Very	38.6	28.6	30.8	0	57.9	χ^2^(6) = 12.7

Somewhat	40.9	42.9	46.2	60	31.6	p = 0.302

Not at all	20.5	28.6	23.1	40	10.5	

Price willing to pay for one package ($)						

Median	5	3	3	2	5	χ^2^(3) = 11.81

(IQR)	(3.00-5.75)	(2.00-3.00)	(2.50-5.00)	(1.00-4.00)	(5.00-11.00)	p < 0.001

* Likelihood variables tested by Pearson chi-square; price tested by Kruskal-Wallis

#### Patterns of Use

**Figure 3:**
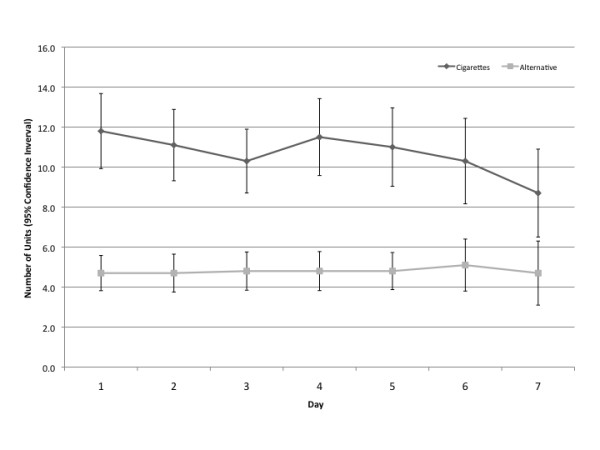
**Alternative product versus cigarette patterns of use during (single-product trial (N = 44) phase**.

As illustrated in Figure 3, over the course of the seven-day trial phase, daily cigarette use decreased from 11.8 to 8.7 cigarettes on average (a 25% reduction; p = 0.004), while alternative product was relatively stable (4.7 units at day 1 to 4.7 units at day 7; p = 0.777). No participant stopped using cigarettes completely. Table 3 shows results of GEE models controlling for gender, age, level of education, HSI score, and alternative product chosen. We observed a significant effect of day [p = 0.004], indicating an overall linear decline in cigarettes smoked per day across the week. Cigarette use across the week was also related to education, with smokers having less than HS education smoking significantly more cigarettes than those with more than HS education. Age was also associated with cigarette use, with those aged 40 or less smoking fewer cigarettes than those aged 55 or higher. Alternative product preferred was not associated with cigarette use during the trial week. In contrast, GEE models showed no significant change in alternative product use across the week, indicating stability in use. However, HSI was positively associated with alternative product use during the trial week--those with higher scores used more units of their chosen product. We also noted an overall effect of education [Wald χ^2^(2) = 13.828, p = 0.001], wherein those had less than HS education used fewer alternative products than those with more than HS education. No statistically significant relationships between alternative product use and gender, age, or preferred product were noted; model adjusted mean use for those who preferred Camel Snus was 3.0 units/day over the week, compared to 3.9 units/day for Marlboro Snus, 4.5 units/day for Commit, and 2.7 units/day for Stonewall.

### Biomarkers of exposure

Exhaled CO decreased significantly from before to after the one-week trial. Exhaled CO before any use of alternative products (Session 2) averaged 18.7 ppm (SD 7.0), and dropped after the one week trial (Session 4) to an average of 14.9 ppm (SD 7.2), a decline of 10% [t(43) = 4.149; p < 0.001]. Overall, 75% of participants demonstrated a decrease in breath CO levels. Geometric mean salivary cotinine was observed to remain stable; at Session 2 saliva cotinine was 311.0 ng/mL, compared to 311.9 ng/mL at Session 4 [t(41) = -0.043; p = 0.966]. We calculated a compensation index for cotinine relative to CO using the formula COMP = 1 - (ln (COT4) - ln(COT2))/(ln(CO4) - ln(CO2)). The median compensation score was 1.02 (IQR 0.38-1.60), suggesting that on average, smokers in the study compensated completely for their nicotine needs by substituting alternative products. Compensation index did not differ by preferred product [Kruskal-Wallis χ^2^(3) = 0.76, p = 0.860].

## Discussion

Findings from this study show that smokers who are currently unwilling to make a quit attempt may be open to adopting alternative products in the short term as a temporary substitute for smoking. The two-step trial period allowed us to explore smokers' willingness to try unfamiliar oral nicotine and smokeless tobacco products, as well as their perceptions and acceptability of these products as an alternative to cigarettes. Data suggest that the dominant behavioral pattern was partial substitution, with about twice as many cigarettes consumed compared to oral nicotine products. Although, we observed that cigarette use declined by nearly one-half during the monitoring interval. We did not collect data to inform whether low use of the alternatives was a result of participants 'rationing' product, but given the number of unused portions returned, this was likely not a major problem. It is more likely that participants had not settled on a habitual use pattern, and/or the products were not sufficiently attractive.

Concomitant measurement of biomarkers of exposure revealed a small but significant decrease in breath CO associated with use of alternative products, without any change in salivary cotinine levels. The modest reduction in CO exposure, while statistically significant, is unlikely by itself to qualify as a true reduction in harm. In contrast, the downward trajectory of cigarette use, paired with consistent use of alternative products, suggests that *ad libitum *use of SL/NR might, over a longer time frame, lead to substantial harm reduction; however, this must be evaluated in longer term studies. The finding of a stable level of substitution over the week of use is consistent with the possibility that smokers, at least those with low interest in quitting, may prefer a gradual shift strategy to an immediate changeover model (as occurs with use of NRT to quit). We cannot predict from the current data whether this process would continue and result in complete substitution, ongoing mixed use, a reversion to cigarettes, or complete cessation of nicotine use.

Similar to Shiffman's survey findings,[27] we also found that among smokers willing to try an oral nicotine product more were interested in using a pharmaceutical nicotine product (Commit) compared to three alternative smokeless tobacco products. This finding of willingness to use a nicotine replacement product for temporary substitution is consistent with prior literature showing substantial use of NRT products for reasons other than quitting (e.g., smoking reduction, temporary abstinence) by smokers [39,40] The observed preference for the nicotine lozenge counters an implicit assumption [41,42] that smokers would more likely prefer smokeless tobacco to nicotine products. At the same time, the greater the range of products offered, the greater the proportion of smokers who may find a product they see as a viable substitute for cigarettes. This is consistent with the body of literature [29-34] suggesting that smokers' varied reactions to different products may be informative in themselves, meaning a 'sampling' approach may allow smokers to find an appealing alternative product to cigarettes.

Participants identified Camel Snus as containing the most nicotine and, while it was not preferred by many (14%), a substantial majority (70%) of these reported they were at least somewhat likely to purchase the product in the next year (see Table 4). This observation may be related to the higher 'free' nicotine content in Camel Snus relative to the other products offered. In particular, other investigators have pointed out the discrepancy between Marlboro Snus and Swedish forms in terms of free nicotine [43]. This difference in nicotine may explain the discrepancy in preference between Camel and Marlboro Snus, which might seem superficially equivalent. Moist snuff manufactures have altered free nicotine levels to affect nicotine delivery and appeal to different markets[44,45]. However, for smokers naïve to oral tobacco products, the higher free nicotine content of Camel Snus may initially have been experienced as aversive while the low free nicotine content of Marlboro Snus may have been insufficiently satisfying to sustain use.

The findings from this study are subject to a number of limitations. About 1/3 of initial participants did not complete all phases of this study, reflecting the challenges of multi-visit studies. The limited number of participants also precluded identifying specific demographic predictors of willingness to substitute or patterns of substitution. We chose not to include a comparison group who continued to use only cigarettes, because our primary interest was observing what smokers might do when presented the opportunity to try a different form of nicotine delivery.

In summary, this study reveals that the nicotine lozenge was viewed more favorably than smokeless tobacco products, as a substitute for cigarettes, which counters some claims that ST would be more acceptable to smokers. However, we observed no true switching (i.e., abandoning cigarettes), even though SL and NR products were provided without cost. It is clear that simply informing smokers of the lower risk and providing products is not going to result in major immediate shifts to smokeless alternatives. In the absence of some significant incentive, it is unlikely that information campaigns alone would lead to migration from use of cigarettes toward less hazardous nicotine sources among United States smokers. Further work is needed of the longer-term effects on attempts at substitution to see if potentially significant effects, in public health terms, can be achieved, or whether encouraging smokeless nicotine use is not a viable substitution strategy.

## Competing interests

RJO served as a consultant to the FDA Tobacco Products Scientific Advisory Committee (Tobacco Constituents Subcommittee). KMC has provided expert testimony on behalf of plaintiffs in cases against the tobacco industry.

## Authors' contributions

RJO, RB, KMC, MCM, and MBT designed the study. Data was collected by KJN. RJO, KJN, and MBT prepared the first draft. All authors provided substantive input on analysis and interpretation of data and the revision of the manuscript and have approved the final version of the manuscript.

## References

[B1] Dube SR Asman K Malarcher A Caraballo R Cigarette Smoking Among Adults and Trends in Smoking Cessation - United States, 2008 MMWR 2009 58 1227 1232 19910909

[B2] Stratton K Shetty P Wallace R Bondurant S eds. Clearing the smoke: Assessing the science base for tobacco harm reduction IOM 2001 Washington, DC: National Academy Press Committee to Assess the Science Base for Tobacco Harm Reduction 25057541

[B3] Bates C Fagerstrom K Jarvis MJ Kunze M McNeill A Ramstrom L European Union policy on smokeless tobacco: a statement in favour of evidence based regulation for public health Tob Control 2003 12 360 367 10.1136/tc.12.4.360 14660767 PMC1747769

[B4] Kozlowski LT Strasser AA Giovino GA Erickson PA Terza JV Applying the risk/use equilibrium: use medicinal nicotine now for harm reduction Tob Control 2001 10 201 203 10.1136/tc.10.3.201 11544374 PMC1747574

[B5] Levy DT Mumford EA Cummings KM Gilpin EA Giovino G Hyland A The relative risks of a low-nitrosamine smokeless tobacco product compared with smoking cigarettes: estimates of a panel of experts Cancer Epidemiol Biomarkers Prev 2004 13 2035 2042 15598758

[B6] Sweanor D Alcabes P Drucker E Tobacco harm reduction: how rational public policy could transform a pandemic Int J Drug Policy 2007 18 70 74 10.1016/j.drugpo.2006.11.013 17689347

[B7] Zeller M Hatsukami D Strategic Dialogue on Tobacco Harm Reduction Group The Strategic Dialogue on Tobacco Harm Reduction: a vision and blueprint for action in the US Tob Control 2009 18 324 332 10.1136/tc.2008.027318 19240228 PMC4915216

[B8] Hatsukami DK Lemmonds C Tomar SL Smokeless tobacco use: harm reduction or induction approach? Prev Med 2004 38 309 317 10.1016/j.ypmed.2003.10.006 14766113

[B9] Savitz DA Meyer RE Tanzer JM Mirvish SS Lewin F Public health implications of smokeless tobacco use as a harm reduction strategy Am J Public Health 2006 96 1934 1939 10.2105/AJPH.2005.075499 17018821 PMC1751814

[B10] Tomar SL Fox BJ Severson HH Is smokeless tobacco use an appropriate public health strategy for reducing societal harm from cigarette smoking? Int J Environ Res Public Health 2009 6 10 24 10.3390/ijerph6010010 19440266 PMC2672338

[B11] Warner KE Tobacco harm reduction: promise and perils Nicotine Tob Res 2002 4 S61 S71 10.1080/1462220021000032825 12580158

[B12] Carpenter CM Connolly GN Ayo-Yusuf OA Wayne GF Developing smokeless tobacco products for smokers: an examination of tobacco industry documents Tob Control 2009 18 54 59 10.1136/tc.2008.026583 18948390

[B13] Mejia AB Ling PM Tobacco industry consumer research on smokeless tobacco users and product development Am J Public Health 2010 100 78 87 10.2105/AJPH.2008.152603 19910355 PMC2791252

[B14] Rogers JD Biener L Clark PI Test marketing of new smokeless tobacco products in four U.S. cities Nicotine Tob Res 2010 12 69 72 10.1093/ntr/ntp166 19917598 PMC2902908

[B15] Rees VW Kreslake JM Cummings KM O'Connor RJ Hatsukami DK Parascandola M Assessing consumer responses to potential reduced-exposure tobacco products: a review of tobacco industry and independent research methods Cancer Epidemiol Biomarkers Prev 2009 18 3225 3240 10.1158/1055-9965.EPI-09-0946 19959675 PMC2790162

[B16] H.R.1256--111th Congress. Family Smoking Prevention and Tobacco Control Act. H.R.1256 2009 Ref Type: Bill/Resolution

[B17] Timberlake DS Are smokers receptive to using smokeless tobacco as a substitute? Prev Med 2009 49 229 232 10.1016/j.ypmed.2009.07.012 19631684

[B18] Gartner CE Jiminez-Soto EV Borland R O'Connor RJ Hall WD Are Australian smokers interested in using low nitrosamine smokeless tobacco for harm reduction? Tob Control 2010 19 451 6 10.1136/tc.2009.033670 20671083

[B19] Wilson N Borland R Weerasekera D Edwards R Russell M Smoker interest in lower harm alternatives to cigarettes: national survey data Nicotine Tob Res 2009 11 1467 1473 10.1093/ntr/ntp152 19828433 PMC2904262

[B20] Cummings KM Hyland A Giovino GA Hastrup JL Bauer JE Bansal MA Are smokers adequately informed about the health risks of smoking and medicinal nicotine? Nicotine Tob Res 2004 6 S333 S340 10.1080/14622200412331320734 15799596

[B21] Bansal MA Cummings KM Hyland A Giovino GA Stop-smoking medications: who uses them, who misuses them, and who is misinformed about them? Nicotine Tob Res 2004 6 S303 S310 10.1080/14622200412331320707 15799593

[B22] Shiffman S Ferguson SG Rohay J Gitchell JG Perceived safety and efficacy of nicotine replacement therapies among US smokers and ex-smokers: relationship with use and compliance Addiction 2008 103 1371 1378 10.1111/j.1360-0443.2008.02268.x 18855827

[B23] Peiper N Stone R van Zyl R Rodu B University faculty perceptions of the health risks related to cigarettes and smokeless tobacco Drug Alcohol Rev 2010 2 121 130 10.1111/j.1465-3362.2009.00143.x 20447218

[B24] Tomar SL Hatsukami DK Perceived risk of harm from cigarettes or smokeless tobacco among U.S. high school seniors Nicotine Tob Res 2007 9 1191 1196 10.1080/14622200701648417 17978994

[B25] O'Connor RJ Hyland A Giovino GA Fong GT Cummings KM Smoker awareness of and beliefs about supposedly less-harmful tobacco products Am J Prev Med 2005 29 85 90 16005803 10.1016/j.amepre.2005.04.013

[B26] O'Connor RJ McNeill A Borland R Hammond D King B Boudreau C Smokers' beliefs about the relative safety of other tobacco products: findings from the ITC collaboration Nicotine Tob Res 2007 9 1033 1042 17943619 10.1080/14622200701591583

[B27] Shiffman S Gitchell J Rohay JM Hellebusch SJ Kemper KE Smokers' preferences for medicinal nicotine vs smokeless tobacco Am J Health Behav 2007 31 462 472 17555377 10.5555/ajhb.2007.31.5.462

[B28] Heavner KK Rosenberg Z Phillips CV Survey of smokers' reasons for not switching to safer sources of nicotine and their willingness to do so in the future Harm Reduct J 2009 6 14 10.1186/1477-7517-6-14 19573235 PMC2714508

[B29] Carpenter MJ Gray KM A pilot randomized study of smokeless tobacco use among smokers not interested in quitting: changes in smoking behavior and readiness to quit Nicotine Tob Res 2010 12 136 143 10.1093/ntr/ntp186 20053788 PMC2816197

[B30] Schneider NG Olmstead RE Nides M Mody FV Otte-Colquette P Doan K Comparative testing of 5 nicotine systems: initial use and preferences Am J Health Behav 2004 28 72 86 14977161 10.5993/ajhb.28.1.8

[B31] Schneider NG Terrace S Koury MA Patel S Vaghaiwalla B Pendergrass R Comparison of three nicotine treatments: initial reactions and preferences with guided use Psychopharmacology (Berl) 2005 182 545 550 10.1007/s00213-005-0123-3 16133134

[B32] Schneider NG Cortner C Justice M Gould JL Amor C Harman N Preferences among five nicotine treatments based on information versus sampling Nicotine Tob Res 2008 10 179 186 10.1080/14622200701767837 18188758

[B33] Caldwell B Burgess C Crane J Randomized crossover trial of the acceptability of snus, nicotine gum, and Zonnic therapy for smoking reduction in heavy smokers Nicotine Tob Res 2010 12 179 183 10.1093/ntr/ntp189 20064899

[B34] Cobb CO Weaver MF Eissenberg T Evaluating the Acute Effects of Oral, Non-combustible Potential Reduced Exposure Products Marketed to Smokers Tob Control 2010 19 367 73 10.1136/tc.2008.028993 19346218 PMC3207996

[B35] Kotlyar M Mendoza-Baumgart MI Li ZZ Pentel PR Barnett BC Feuer RM Nicotine pharmacokinetics and subjective effects of three potential reduced exposure products, moist snuff and nicotine lozenge Tob Control 2007 16 138 142 10.1136/tc.2006.018440 17400953 PMC2598476

[B36] Richter P Spierto FW Surveillance of smokeless tobacco nicotine, pH, moisture, and unprotonated nicotine content Nicotine Tob Res 2003 5 885 889 10.1080/14622200310001614647 14668072

[B37] Stepanov I Jensen J Hatsukami D Hecht SS New and traditional smokeless tobacco: comparison of toxicant and carcinogen levels Nicotine Tob Res 2008 10 1773 1782 10.1080/14622200802443544 19023828 PMC2892835

[B38] Heatherton TF Kozlowski LT Frecker RC Rickert W Robinson J Measuring the heaviness of smoking: Using elf-reported time to the first cigarette of the day and number of cigarettes smoked per day Br. J. Addiction 1989 84 791 800 10.1111/j.1360-0443.1989.tb03059.x 2758152

[B39] Levy DE Thorndike AN Biener L Rigotti NA Use of nicotine replacement therapy to reduce or delay smoking but not to quit: prevalence and association with subsequent cessation efforts Tob Control 2007 16 384 389 10.1136/tc.2007.021485 18048614 PMC2807189

[B40] Hammond D Reid JL Driezen P Cummings KM Borland R Fong GT Smokers' use of nicotine replacement therapy for reasons other than stopping smoking: findings from the ITC Four Country Survey Addiction 2008 103 1696 1703 10.1111/j.1360-0443.2008.02320.x 18821877 PMC4605436

[B41] Gartner C Hall W Harm reduction policies for tobacco users Int J Drug Policy 2010 21 129 130 10.1016/j.drugpo.2009.10.008 19944582

[B42] Kozlowski LT Effect of smokeless tobacco product marketing and use on population harm from tobacco use policy perspective for tobacco-risk reduction Am J Prev Med 2007 33 S379 S386 10.1016/j.amepre.2007.09.015 18021913

[B43] Foulds J Furberg H Is low-nicotine Marlboro snus really snus? Harm Reduct J 2008 5 9 18304348 10.1186/1477-7517-5-9 PMC2288606

[B44] Alpert HR Koh H Connolly GN Free nicotine content and strategic marketing of moist snuff tobacco products in the United States: 2000-2006 Tob Control 2008 17 332 338 10.1136/tc.2008.025247 18669556

[B45] Tomar SL Henningfield JE Review of the evidence that pH is a determinant of nicotine dosage from oral use of smokeless tobacco Tob Control 1997 6 219 225 10.1136/tc.6.3.219 9396107 PMC1759570

